# Neurological Soft Signs and Post-Traumatic Stress Disorder: A Biomarker of Severity?

**DOI:** 10.3389/fpsyt.2020.533662

**Published:** 2020-10-20

**Authors:** Célia Belrose, Anais Duffaud, Elsa Rakotoarison, Catherine Faget, Philippe Raynaud, Frédéric Dutheil, Léa Boyer, Jean-Baptiste Billaud, Marion Trousselard

**Affiliations:** ^1^ Département de Neurosciences et Sciences Cognitives, Unité de Neurophysiologie du Stress, Institut de Recherche Biomédicale des Armées, Brétigny sur Orge, France; ^2^ Réseau ABC des Psychotraumas, Montpellier, France; ^3^ APEMAC, EA 4360, Université de Lorraine, Nancy, France; ^4^ Hôpital la Conception, APHM, Marseille, France; ^5^ Centre Hospitalier Léon Jean Grégory, Thuir, France; ^6^ Université Clermont Auvergne, CNRS, LaPSCo, Physiological and Psychosocial Stress, CHU Clermont-Ferrand, Clermont-Ferrand, France; ^7^ Faculty of Health, School of Exercise Science, Australian Catholic University, Melbourne, VIC, Australia

**Keywords:** recovery, neurological soft signs, post-traumatic stress disorder (PTSD), cerebellum, gender

## Abstract

**Background:**

The psychophysiological changes for individual suffering from chronic post-traumatic stress disorder (PTSD) raise to the questions of how facilitate recovery and return to work. Negative alterations in neuro-cognition remain a complaint for patients and participate to long-term functional impairments. Neurological soft signs (NSSs) appear as a candidate for better understanding these complaints. They have been reported in several mental disorders. They are found in several behavioral and/or neurocognitive disorders and are taken into account by psychiatric rehabilitation programs to support recovery. As few studies evaluate NSSs in PTSD, our exploratory study aims to assess NSSs in chronic PTSD and their relationships with PTSD severity.

**Method:**

Twenty-two patients with a clinical diagnosis of chronic PTSD were evaluated in terms of PTSD severity (post-traumatic checklist scale, PCL5), NSSs (NSSs psychomotor skills scale, PASS), and well-being upon arrival to the hospital and compared with 15 healthy subjects. Statistical non-parametric analyses assessed the relationships between these variables.

**Results:**

PTSD subjects exhibited higher NSSs compared with healthy subjects. NSSs were positively associated with PTSD severity, with negative alterations in cognition and mood, and with impairment in well-being. They were higher in women compared with men. No impact of age was found. Three groups were identified based on the severity of the PTSD. Severe PTSD exhibited NSSs characterized by motor integration alterations.

**Conclusions:**

This pilot study suggests that NSSs might be a biomarker of PTSD severity. This proof of concept highlights the need for further research for better evaluating the clinical neuro-functional impairment. This will be helping for defining neurological remediation for promoting PTSD recovery.

## Introduction

### Post-Traumatic Stress Disorder

Post-traumatic stress disorder (PTSD) is a debilitating mental disorder that may develop after experiencing or witnessing a life-threatening event. The main characteristics of PTSD are re-experiencing symptoms, avoiding situations that recall the event, negative alterations in cognition and mood and hyperarousal (1). PTSD is associated with impairment in social, occupational and other domains (2) for at least one month. Once those symptoms have been observed for 3 months, PTSD is considered as a chronic disorder (3). Furthermore, the prevalence of comorbidities is high including: depression, substance use disorders and general physical health effects (4, 5).

The lifetime PTSD prevalence was found to range from 1% to 7% in Europe (5). According to the country and the type of trauma, the mean 12-month prevalence of PTSD is between 25% and 50% (6). Among the military, PTSD prevalence is highly dependent on the violence of the mission; the higher the combat exposure is, the higher PTSD prevalence is (up to 20%) (7).

Despite appropriate care, treatment response is variable and almost 20% of the patients do not show condition’ improvement. This variability in response treatment arises from (i) the lack of research in precision medicine (i.e., which treatment suits best the patient) and (ii) the nature of the physiopathology of the PSTD (8). In a 20-year longitudinal study conducted on 214 veterans with initial combat stress reaction, Solomon and Mikulincer (2006) highlighted the volatility of chronic stress with a relapse observed in 40% of the recovering subjects within one year of remission (9).

Currently available tools for assessing prognosis when PTSD are inadequate. Impairments in emotional, self-regulatory, and cognitive functions are biomarkers of interest when regarding their critical influence on post-traumatic processing, treatment efficiency and recovery. PTSD has been associated with negative emotions (10), disturbed positive resources (11), as well as impairments in the ability to effectively regulate emotions (12). Moreover, a number of prospective studies indicate that emotional regulation difficulties hinder recovery from PTSD symptoms post-trauma (13).

Putative neural mechanisms underlying the PTSD symptoms involve altered brain regions including the hippocampus and amygdala as well as cortical regions including the anterior cingulate, insula, and orbitofrontal regions (14, 15). These brain alterations have an impact on connection to neural circuits that mediates adaptation to stress and fear conditioning. Altogether, these brain disorders have been proposed to have a direct link to the emotional, self-regulatory, and cognitive impairments in PTSD (16).

Despite advances in the understanding of the neural circuitry associated with emotional, self-regulatory impairments and how they impact prognosis by disturbing treatment outcomes, there is a paucity of research investigating the global cognitive dysfunctions in PTSD. Nevertheless, cognitive deficits are one of the most consistent predictors of chronic disability found among both younger and older people with psychiatric illness (17–19). It is well-known that PTSD is associated with decrements in multiple cognitive systems, including processing speed, learning, memory, and executive function (20–22) and inability to divert attention away from trauma-provoking stimuli (21, 23, 24). Such deficits are well-known to increase the effects of psychosocial stressors related to physical health behaviors (25), academic and vocational productivity (26), and interpersonal relationships (27).

Interestingly, literature reports associations of neurological soft signs (NSSs) with poor cognitive performance in healthy subjects (28–31) as well as in patients with chronic psychiatric diseases, such as schizophrenia (32, 33), and bipolar subjects (34, 35). NSSs are objective performance measures of sensorimotor, reflexive, perceptual, and cognitive capabilities. They reflect minor neurological abnormalities thought to be manifestations of a minor nonspecific cerebral dysfunction (either localized or diffuse) which yield subtle indicators of brain dysfunctions (30). NSSs are different from the “hard” neurological signs. The latter are often indicative of a basic sensory or motor deficit that is considered to be directly related to an injury to a specific brain region. Recent studies using non-clinical samples have suggested that neural correlates of a cognitive function are likely to be distributed throughout the brain rather than localized to specific areas (36, 37). A similar proposal has been considered for NSSs (30), NSSs have been found to be elevated in a variety of mental disorders, with more than 100 studies of NSSs in the psychiatric literature (Pubmed search). A meta-analysis establishes a link between the NSSs and the biological markers of psychiatric vulnerability (38). This shows the interest of NSSs for prognosis of a psychiatric disease all the more that examination of NSSs entails low-tech, inexpensive, relatively brief, and readily administered clinical maneuvers. Furthermore, studies with bipolar suffering suggest that NSSs would progress only minimally with increasing age (35).

Although NSSs have been less investigated in PTSD, they were reported in male veterans with chronic combat-related PTSD (but not in combat-exposed veterans without PTSD) as in adult females with PTSD as a result of childhood sexual abuse (39). In these populations, NSSs were associated with more reported neurodevelopmental problems, e.g., attention deficit, motor hyperactivity, and learning problems (39). However, NSSs were not found in the veteran nurses with PTSD (40). These conflicted results question the role of premorbid neurodevelopment as a risk factor for NSSs in PTSD when traumatic exposure. They also highlight three major gaps in the literature regarding the role of NSSs in PTSD: (1) the pertinence of NSSs as a biomarker of PTSD severity and prognosis, (2) the domain specificity of the involvement of NSSs in cognitive performance, and (3) whether there is sex difference.

This study aims to be a proof of concept in the exploration of the relationship between PTSD severity and NSSs.

## Materials and Methods

### Participants

Two groups of voluntary civilian subjects were recruited: 22 patients suffering from PTSD through the psychiatric consultation in public hospitals of Marseille and Perpignan (France) and 15 healthy subjects matching according to age and gender through the personal from hospitals of Marseille. This study received the agreement of the ethics committee of the French military health service (MHS). After a complete description of the study, written informed consent for participation in this low-risk study was obtained.

### Protocol

Informed and volunteer patients with at least one positive response in the criterion A as described in the DSM5 and clinically evaluated by a psychiatrist with a diagnosis of chronic PTSD (more than 6 months since the first psychiatrist PTSD diagnosis without improvement) were included in the study. Self-reported left-handed was a non-inclusion criterion. After their consultation, patients were screened with the following assessments: PTDS severity, well-being and NSSs. These assessments lasted 2h and 30 min.

Informed and volunteer control subjects

### Variables

For each subject, the collected socio-demographic included: age, gender, and the number of major stresses encountered in professional and personal environments over subject lifetime.

NSSs were also evaluated using Psychomotor Assessment of the Sweet Signs (PASS) [(41); http://www.psychomot.ups-tlse.fr/EPSID.pdf]. This hetero-questionnaire was developed using the available scale for assessing the NSSs. It was validated in a French population of children and adults. Processing the PASS requires very few materials. Only the test of stereognosies requires the use of the following equipment: a key, a coin, a button, a battery, a dice, a clothespin. Twenty-seven tasks were evaluated and combined in 9 categories of NSSs: (i) walking evaluation implying different types of walking (tasks 1 to 5), (ii) static and dynamic equilibrium (tasks 6 to 8), (iii) perseverance in task (task 9), (iv) tonus (tasks 12 to 15), (v) motor integration included complex and simple sequences of motor coordination (tasks 10, 11, and 19 to 25), (vi) sensory integration (tasks 28–30), (vii) dysrhythmias (tasks 6, 10, 11, and 20 to 25), (viii) synkinesis (tasks 10 and 20–25), and (ix) somatognosis and spatial self-perception (tasks 26–27). A total score is calculated from the 27 tasks. Scores range from 0 (no NSS) to 135 (maximum errors for each evaluated task). An evaluation of manual laterality coefficient (handedness) was realized at the end of the PASS. No cut-off has been validated for this scale.

#### For PTSD Subjects (PTSD Group)

In addition to the PASS, patients completed the two following questionnaires.

The auto-questionnaire used to assess PTSD severity was the PTSD Check List Scale (PCL-5) (1, 42). It assesses the following four symptoms: re-experiencing symptoms, avoiding situations that recall the event, hyperarousal and impairment of cognitive and emotional affects. Higher scores indicate higher severity. The cut-off point proposed by the National Center for PTSD is a score above or equal to 33.

Well-being was assessed using the Warwick-Edinburgh Mental Well-Being Scale (WEMWBS) (43, 44). This auto-questionnaire covers both affective constructs (including the experience of happiness) and constructs representing psychological functioning and self-realization (45). This is a 14-item scale on thoughts and feelings over the past week; each item ranges from (1) “none of the time” to (5) “all of the time”. Higher scores indicate higher well-being. No cut-off has been validated for this scale.

#### For Healthy Subjects (Control Group)

In addition to the PASS, healthy subjects were assessed by a psychiatrist using the structured Mini-International Neuropsychiatric Interview (46) to check for the absence of a psychiatric disorder and to screen for potential psychiatric disorders.

### Statistical Analysis

Data analyses were performed using the Statistica (Stastsoft France, Maison Alfort, v7.1) software.

The values are expressed as mean ± standard error of mean. Correlations were done using Bravais-Pearson analyses. Comparisons between groups were performed using Pearson’s chi-square test for variables with several modalities and using t-test for the quantitative data or nonparametric Kruskal-Wallis analyses as they did not have a normal distribution.

For the PTSD group, we characterized patients according to PTSD diagnosis and severity. Three groups were defined according to the PCL5 score: a group with a score under 33 (group with minimal PTSD; Minor PTSD), a group with a score under the median of the PCL5 score of our population (group with moderate PTSD; Moderate PTSD), and a group with a score above the median of the PCL5 score of our population (group with severe PTSD; Gr Severe PTSD). Between group comparisons were performed using Kruskal-Wallis test followed by Dunn’s *post-hoc* test (including Bonferroni correction) (47, 48). The statistical threshold of significance was set at p < 0.05. The trends are taken in consideration when p < 0.10.

## Results

### Population Description

For the PTSD group, 22 patients were included in the study, 16 females (72.73%) and 6 males (27.27%). They were on average 37.86 (± 12.74) years old aged with a median age of 34.5. The clinical evaluation confirmed PTSD diagnosis for each subjects.

Average score of the clinical severity at PCL5 was 52.35 (± 19.8) and the median value of scores of the PCL5 was 55 (range: 15–84). Negative correlations were found between age and PCL5 score (r^2^ = −0.41, p = 0.06), as between PCL5 score and negative mood and cognition (r^2^ = −0.48, p = 0.028) and hyperarousal (r^2^ = −0.47, p = 0.03) sub-scores.

Average score of the WEMWBS was 47.77 (± 15.15) and the median value of scores of the PCL5 5 was 51.5 (range: 28–60). Negative correlations were found between WEMWBS and PCL5 scores (r^2^ = −0.61, p = 0.003), as between WEMWBS and negative alterations in cognitions and mood (r^2^ = −0.71, p < 0.001) and hyperarousal (r^2^ = −0.57, p = 0.007) sub-scores. No correlation was found between age and WEMWBS score (r^2^ = 0.25, p = 0.26).

Concerning the PASS evaluation, the laterality coefficient showed that 18 (81.81%) of the patients were right-handed among which 68.18% of the patients had a right handedness coefficient of 100. Three (13.63%) had a coefficient between 20 and 80; one subject (4.54%) had a coefficient of −30, meaning that this patient was left-handed. Average score of the NSSs was 14.04 (± 15.15) and the median value of scores of the PASS is 6.5 (range: 1–45.75) (**Table 1**). Positive correlations were found between PASS and PCL5 scores (r^2^ = 0.69, p < 0.001), as between PASS score and intrusions (r^2^ = −0.48, p = 0.028), negative alterations in cognitions and mood (r^2^ = −0.59, p = 0.005) and hyperarousal (r^2^ = −0.73, p < 0.001) sub-scores. A negative correlation was found between PASS and WEMWBS scores (r^2^ = −0.36, p = 0.10). No correlation is found between age and WEMWBS score (r^2^ = −0.16, p = 0.46).

**Table 1:** Mean scores, standard deviations (±), medians, and ranges for the main items of the PASS with significant gender effect, with left or right precision when necessary.

		My ± ET	Median	RangeMin-Max	Gender effectp
NSSs total score		14.04 ± 15.15	6,5	1–45.75	0.007
Spontaneous tempo (s)		11.03 ± 2.51	11	6.83–14.86	0.028
Fastest tempo (s)		7.05 ± 3.24	5.93	3.02–13	
Walking	Gait	0.26 ± 0.54	0	0–2	
	Gait on tiptoes	0.40 ± 0.66	0	0–2	
	Gait on heels	0.71 ± 0.78	1	0–2	
	Gait with inversion/eversion	1.52 ± 1.57	1	0–5	
	Gait with heel-and-toe	1.14 ± 1.42	0	0–4	
Static and dynamic equilibrium	Jumping up and down on one foot*	1.62 ± 2.42	0.5	0–8	
	Lowest time of unipodal equilibrium (s)**	0.35 ± 0.75	0	0–3	
	Average time of unipodal equilibrium (s)**	35.71 ± 24.21	28.25	3.83–71	0.038 Right 0.077 Left
	Romberg stability*	0.57 ± 0.73	0	0–2	
Protrusion of the tongue	Average of time (s)				
	Abnormal movement (yes or not)				
	Diadochocinesia (s)*	8.47 ± 1.27	0	2.44–21	
	Average time of arm in horizontal position (EO)*	7.88 ± 2.63	8.50	3.01–13.5	0.060 Right
	Opening and closing hands	0.38 ± 0.74	0	0–2	
	Finger-nose pointing EO/EC	0.19 ± 0.43/ 0.36 ± 0.67	0/0	0–1.5/0–2	
	Average time of tapping between thumb and index finger	12.00 ± 4.92	11	4–21.85	0.07 Right
	Tapping	0.68 ± 1.22	0	0-4	0.072 Right
	Average time of thumb fingers (s)	8.99 ± 4.23	9	4–20.5	
	thumb fingers opposition	0.83 ± 1.37	0	0–5	
	Average time of 5 sequences of fist-side-palm	1.84 ± 2.50	0.25	0–8	
	To tap the feet	0.35 ± 0.81	0	0–3	0.063 Left
	Heel-and-toe tap	0.74 ± 1.26	0	0–4	0.09 Right
Sensory integration	Sterognosis (EC)	0.09 ± 0.27	0	0–1	
	Extinction (EC)	0.33 ± 0.59	0	0–2	
	Graphestesia	0.19 ± 0.44	0	0–1.5	
Tonus	Tonicity of upper limb	0.02 ± 0.11	0	0–0.5	
	Upper limb dangling	0.29 ± 0.64	0	0–2	
	Tonicity of lower limb	0.25 ± 0.55	0	0–1.75	
	Lower limb dangling	0.08 ± 0.24	0	0–1	
Somatognosis and spatial perception	Self-right and left evaluation				
	Other-right and left evaluation	0.45 ± 0.83	0	0–3	

EO, eyes opened; EC, eyes closed. *left and right average. **assessment for dominance laterality. Gray lines indicate that tasks are performed correctly for all subjects with a null score.

A gender effect was observed for the NSSs with higher NSSs total score and some of the sub-scores (or tendencies) for females (**Table 1**). Females exhibited higher PCL5 score (W = 16, p = 0.2), with higher alterations in arousal and reactivity (W = 7, p = 0.003) sub-score, and a trend to higher intrusions (W= 24, p = 0.08) sub-score. No gender difference was not found for WEMWBS score (W= 66.5, p = 0.18).

No difference was found between the two centers for each of the scales’ score.

For the control group, 15 healthy subjects were included, 8 females (53.33%) and 7 males (46.67%). They were on average 35.8 (± 11.11) years old aged with a median age of 30. The clinical evaluation from the MINI confirmed the absence PTSD and other psychopathological diagnosis for each of the subjects. The number of traumatic life event was zero for each of the subjects.

Average score of the NSSs was 1.56 (± 1) and the median value of scores is 1.92 (range: 0–3.27). Control group exhibited a lower NSSs average total score than PTSD group (t = 2.65, p = 0.01).

### Impact of PTSD Severity

According to the PCL5 cut-off of 33, five patients (22.72%) were not positive for the psychometric PTSD diagnosis (group called minor-PTSD). No difference was found between the two centers for the number of patients under the cut-off. For the remaining patients with a score above the cut-off, we defined two groups according to the PCL5-median of 58 for these patients; the group called moderate-PTSD consisted of 8 patients (36.36%) with a PCL5 score under 58 and the group called severe-PTSD consisted of 9 patients (40.91%) above or equal to 58.

The three groups differed in terms of age (H = 7.162; p = 0.027) with the highest average age for the minor-PTSD group (51.6 ± 13.83) compared to the moderate- and severe-PTSD groups (33.25 ± 9.88, and 34.33 ± 9.59, respectively). Difference was observed in terms of gender between groups (X^2^ = 6.49, p = 0.04) with the highest number of women in the severe-PTSD group (100%; 9 women) compared to the moderate-PTSD group with 62.5% of women (5 women) and the minor-PTSD group with 40% of women (2 women). No difference was observed for marital status, and the number of stressor in professional as personal life between the three groups.

**Table 2** described the significant differences (or trends) between the three groups for the PCL5, the WEMBS, and the PASS scores and sub-scores. Patients with severe PTSD exhibited the highest symptoms scores, the lowest well-being score and minor-PTSD patients exhibited the lowest symptoms scores, the highest well-being score, and the patients with moderate PTSD were in between. For the significant NSSs differences, the alterations were maximum for the patients with severe PTSD, minimum for minor PTSD patients and in-between for the patients with moderate PTSD. For both the average time of unipodal equilibrium (s) and fastest tempo, alterations were not different between moderate and severe PTSD.

**Table 2:** Mean scores and standard deviations (±) according to the three groups for the items of the PCL5, WEMWBS, and PASS with significant group differences.

			Minor PTSDn = 5	Moderate PTSDn = 8	Severe-PTSDn = 9	Group effectp
PCL5		Total	23.4 ± 6.88	50.5 ± 6.89	71.6 ± 10.1	<0.01^a^
		Intrusion	11.6 ± 3.85	13.62 ± 2.72	17.6 ± 5.33	0.054^a^
		Avoidance	3.87 ± 1.3	5.5 ± 1.85	6.9 ± 2.38	0.064^a^
		Negative mood and cognition	2 ± 2.92	15.87 ± 6.24	25.6 ± 4.72	<0.001^a^
		Alterations in arousal and reactivity	6 ± 2.83	15.5 ± 4.63	21.5 ± 3.5	<0.001^a^
WEMWBS			55.2 ± 2.39	51 ± 8.37	40.78 ± 8.64	0.011^a^
NSSs total score			2.06 ± 2.19	12.06 ± 11.5	23.15 ± 18.1	0.022^a^
Fastest tempo (s)			10.6 ± 2.07	5.39 ± 2.84	6.48 ± 2.65	0.020^b^
Static and dynamic equilibrium	Jumping up and down on the right foot		0	0.25 ± 0.53	0.7 ± 0.57	0.036 R^a^
	Average time of unipodal equilibrium (s)*		30 ± 11.54	18.98 ± 17	18.71 ± 18.73	0.023^b^
	Movements during Romberg		0 0	0.06 ± 0.18 0.06 ± 0.18	0.29 ± 0.27 0.25 ± 0.27	0.053 R^a^ 0.086 L^a^
motor integration and coordination	Average time for 10 prosupinations sequences (s)		11 ± 4.24 12 ± 4.74	5.29 ± 2.41 5.43 ± 1.99	8.53 ± 5.82 9.27 ± 6.43	0.081 R^a^ 0.057 L^a^
	Average time for arm in horizontal position (EO) (s)		10.2 ± 2.17 10.2 ± 1.64	6.7 ± 1.43 6.78 ± 1.4	7.54 ± 3.31 7.6 ± 3.44	0.058 R^a^ 0.03 L^a^

^a^Differences (or trends) between the three groups; ^b^low PTSD differed (or trended to differ) from moderate and severe PTSD the three groups.

EO, eyes opened; EC, eyes closed. *dominance laterality. R, right task; L, left task.

## Discussion

This study explored the relationship between PTSD severity and NSSs as a proof of concept.

According to the three major gaps in the literature, main results highlight that (1) NSSs level reflects the severity of PTSD, (2) the prominent NSSs associated with PTSD are related to static and dynamic equilibrium (i.e., motor integration and coordination), and (3) NSSs primarily affect women.

First, PTSD patients exhibited higher NSSs compared with healthy control subjects. Concerning the relationship between PTSD severity and NSSs, results suggest that the alterations in NSSs increase with PTSD severity. Regarding our results, minor PTSD with the lowest NSSs are the oldest patients. This data is not sufficient to affirm that age does not interact with NSSs and further studies are needed to evaluate this relationship, namely, prospective data are needed for describing the co-evolution of clinical symptoms and NSSs. Furthermore, there is some evidence suggesting that some inflammatory mechanism would be a mediator between PTSD clinical severity and NSS (49, 50). An increasing number of studies examining PTSD have either emphasized a relationship between PTSD and a systemically pro-inflammatory state or identified a link between PTSD and chronic disease. Namely, PTSD symptoms constitute a stress-perpetuating syndrome that maintains the individual in a chronic state of sustained stress (51, 52). Emerging evidence suggests that the biological consequences this include elevated systemic levels of inflammation implying in accelerated cellular aging and neuroprogression (52). Consequently, the inflammatory pathological remodeling of neural circuitry should occur over the course of a chronic mental illness. Literature questions how inflammatory processes and chronic disease issues are interrelated: putative causes for inflammation in PTSD and possible consequences of inflammation in this disorder (49). The scarcity of longitudinal data does not establish whether the increase in proinflammatory markers precedes or follows the onset of PTSD.

Concerning the type of NSSs associated with PTSD, two prominent alterations were found: static and dynamic equilibrium as well as motor integration and coordination NSSs. Altogether, these NSSs alterations highlight the role of the cerebellum. While the cerebellum has, until recently, not been considered as a key region in PTSD, there is growing evidence implicating the cerebellar region in the pathophysiology of PTSD (53, 54). Convergent findings from neuroimaging and lesion studies showed that the cerebellum’s role is not confined to motor function (55) but is also important in cognition and emotion (56, 57). Thus, some authors proposed that patients with PTSD exhibited alterations in both top-down and bottom-up emotion regulation (53). From the top-down of view, the hypothesis on PTSD is a learned incapacity of top-down structures as prefrontal cortex in inhibition of an “hyper-reactive” amygdala (58, 59). The down-top frame suggests a role of cerebellum deficits to control the hyper-reactivity of amygdala, too (54). Indeed, it is known that the cerebellum receives and sends information to non-motor cortical areas, including prefrontal regions responsible for higher cognitive functions (60) and that both amygdala and cerebellum are crucial sites in fear conditioning (61, 62) and extinction models (63). Furthermore, it has been recently described the relationship. Furthermore, correlations between functional connectivity in the cerebellum, symptoms severity, included the four symptom domains specified in the DSM-5, has been described (54). Altogether, these data led to propose a non-specific role of the cerebellum in PTSD symptomatology with neurological consequences that could be linked to the severity of the PTSD and its prognosis. The left-right asymmetry in the prominent NSSs is difficult to discuss since left and right handedness subjects are included among our cohort. There a need to evaluate the interaction between objective laterality and gender difference in further studies for better describe functional lateralization of the cerebellum (64). This issue is very important and in that respect some studies observed altered functioning of the left cerebellar hemisphere (65) and vermis (66, 67) in PTSD patients. Further studies are needed for better understanding of the cerebellum involvement in the pathophysiology of PTSD. This could helpful for a better evaluation of the relationships between distinct patterns of cerebellar alterations and the clinical severity, included neurological symptoms as for reducing the gap that continue to exist in the understanding of brain structure and function in PTSD.

The issue that NSSs primarily affect women in our study is associated with the highest PTSD severity for the included women. Such results must be considered with caution due to the small size of the sample. Nevertheless, most findings on gender differences in PTSD found that to be a woman is a risk factor for PTSD when trauma: women are approximately twice as likely as men to meet criteria for PTSD following a traumatic event (68), and they are more than four times as likely as men to develop chronic PTSD (69). These data could account for both the 75% women among our PTSD cohort and the disproportionate number of those experiencing severe PTSD. Interestingly, across various studies, women are about one-third less likely than men to report having experienced a trauma (69, 70). These results suggest that the higher rate of PTSD among women cannot be attributed to a greater overall risk of trauma but to a greater vulnerability to PTSD (70). While studies delineate more precisely the ways in which culture, and gender role, alone and in combination shape the gender differences of PTSD (71), neurobiological mechanisms may account for why women reported PTSD more often than men after a trauma. To date, most researchers in this area primarily paid attention to men with only 2% of neurobiological research conducted in females (mainly rats) (69, 70, 72, 73). From a biological point of view, women appear to have a more sensitized hypothalamus–pituitary–axis than men when facing a stressor (69). The oxytocin regulation of fear also differs between men and women (74). These findings indicate that females acquire fear more easily than males (75). Furthermore, gender differences were found in some cerebellum structures with less gray matter volume and less hemispherical asymmetry for women (76). In addition, a gender-related difference in the cerebellar-thalamic-cortical circuitry has been found (77). A developmental hypothesis has also been advanced for gender difference in cerebellar structures and functioning (78). Altogether, these issues highlight the importance of considering gender as a biological variable in cerebellum research for better understanding how gender acts as a susceptibility or resilience factor for PTSD.

This exploratory study has several limits. The first one is the small sample size. Then, results are to be considered as a proof of concept for further studies. Especially, the causal relationship between NSSs and PTSD severity need to be studied. The second one focuses on the sociodemographic characteristics of the population. Both objective right-hander and left-hander PTSD were included. Related to the asymmetrical differences observed in the NSSs among our patients, this points the importance to control the laterality using objective evaluation instead of self-report before exploration of the NSSs. Moreover, women are overrepresented in our sample. Related to the gender differences in PTSD, how gender affects the link between NSSs and PTSD severity, and in what specific ways, need to be further evaluated. Third, evaluation of NSSs was done using the only validated French questionnaire. A need for developing objective scales among countries are needed (i) for better understanding how NSSs are involved in PTSD and (ii) further for comparing NSSs among psychiatric disorders. Such tools will be useful for studying NSSs as subtle indicators of brain dysfunctions and their neurological correlates. Finally, no hypothesis was made on the role of the type of trauma as on the role of the time between the trauma and the clinical inclusion. To confirm whether NSSS are in line with the severity, these factors need to be controlled for future investigations.

## Conclusion

This study as a proof of concept highlights the interest of studying NSSs in PTSD. These exploratory results found a relationship between the severity of PTSD and the NSSs in terms of static and dynamic equilibrium and motor integration and coordination but they do not preclude generalizability or causal relationship. Regarding the asymmetrical NSSs and the gender effect, this points out the methodological implication for future studies. They provide some convincing arguments for evaluating the NSSs as a prognosis factor in PTSD using longitudinal follow-up.

## Data Availability Statement

The datasets generated for this study are available on request to the corresponding author.

## Ethics Statement

The studies involving human participants were reviewed and approved by 2019-A01232-37. The patients/participants provided their written informed consent to participate in this study.

## Author Contributions

CB and MT conceived the study. All authors actively took part in the process. All authors have planned and participated in the statistical analysis. All authors contributed to the article and approved the submitted version.

## Funding

The work was supported by grants (5000 euros) from the Army.

## Conflict of Interest

The authors declare that the research was conducted in the absence of any commercial or financial relationships that could be construed as a potential conflict of interest.

## References

[1] American Psychiatric Association Diagnostic and statistical manual of mental disorders. 5th edn. American Psychiatric Publishing (2013).

[2] Holowka DW Marx BG Assessing PTSD-related functional impairment and quality of life. In: Beck GJ, Sloan DM, Eds. Oxford handbook of traumatic stress disorders. New York (NY): Oxford University Press (2011).

[3] Bisson J II Roberts NP Andrew M Cooper R Lewis C Psychological therapies for chronic post-traumatic stress disorder (PTSD) in adults. Cochrane Database Syst Rev (2013) 12, CD003388. 10.1002/14651858.CD003388.pub4 PMC6991463 24338345

[4] Hoge CW Terhakopian A Castro CA Messer SC Engel CC Association of posttraumatic stress disorder with somatic symptoms, health care visits, and absenteeism among Iraq war veterans. Am J Psychiatry (2007) 164(1):150–3. 10.1176/ajp.2007.164.1.150 17202557

[5] Kessler RC Chiu WT Demler O Merikangas KR Walters EE Prevalence, severity, and comorbidity of 12-month DSM-IV disorders in the National Comorbidity Survey Replication. Arch Gen Psychiatry (2005) 62(6):617–27. 10.1001/archpsyc.62.6.617 PMC2847357 15939839

[6] Yehuda R Hoge CW McFarlane AC Vermetten E Lanius RA Nievergelt CM Post-traumatic stress disorder. Nat Rev Dis Primers (2015) 1:15057. 10.1038/nrdp.2015.57 27189040

[7] Kok BC Herrell RK Thomas JL Hoge CW Posttraumatic stress disorder associated with combat service in Iraq or Afghanistan: reconciling prevalence differences between studies. J Nerv Ment Dis (2012) 200(5):444–50. 10.1097/NMD.0b013e3182532312 22551799

[8] NICE (2018). https://www.nice.org.uk/guidance/ng116 Published date: December 2018.

[9] Solomon Z Mikulincer M Trajectories of PTSD: a 20-year longitudinal study. Am J Psychiatry (2006) 163(4):659–66. 10.1176/ajp.2006.163.4.659 16585441

[10] McLean CP Foa EB Emotions and emotion regulation in posttraumatic stress disorder. Curr Opin Psychol (2017) 14:72–7. 10.1016/j.copsyc.2016.10.006 28813323

[11] Belrose C Duffaud A Dutheil F Trichereau J Trousselard M Challenges Associated With the Civilian Reintegration of Soldiers With Chronic PTSD: A New Approach Integrating Psychological Resources and Values in Action Reappropriation. Front Psychiatry (2019) 9:737. 10.3389/fpsyt.2018.00737 30670989 PMC6333022

[12] Chesney SA Gordon NS Profiles of emotion regulation: understanding regulatory patterns and the implications for posttraumatic stress. Cognit Emot (2016) 2016:1–9. 10.1080/02699931.2015.1126555 26743908

[13] Bardeen JR Kumpula MJ Orcutt HK Emotion regulation difficulties as a prospective predictor of posttraumatic stress symptoms following a mass shooting. J Anxiety Disord (2013) 27(2):188–96. 10.1016/j.janxdis.2013.01.003 PMC3628280 23454838

[14] Hayes JP Hayes SM Mikedis AM Quantitative meta-analysis of neural activity in posttraumatic stress disorder. BMAD (2012) 2(1):9. 10.1186/2045-5380-2-9 22738125 PMC3430553

[15] Pitman RK Rasmusson AM Koenen KC Shin LM Orr SP Gilbertson MW Biological studies of post-traumatic stress disorder. Nat Rev Neurosci (2012) 13(11):769–87. 10.1038/nrn3339 PMC4951157 23047775

[16] Rauch SL Shin LM Phelps EA Neurocircuitry models of posttraumatic stress disorder and extinction: human neuroimaging research-past, present, and future. Biol Psychiatry (2006) 60:376–82. 10.1016/j.biopsych.2006.06.004 16919525

[17] Green MF Kern RS Braff DL Mintz J Neurocognitive deficits and functional outcome in schizophrenia: are we measuring the”right stuff”? Schizophr Bull (2000) 26:119–36. 10.1093/oxfordjournals.schbul.a033430 10755673

[18] Heaton RK Pendleton MG Use of neuropsychological tests to predict patients everyday functioning. J Consult Clin Psychol (1981) 49:807–21. 10.1037/0022-006X.49.6.807 7309951

[19] Twamley EW Doshi RR Nayak GV Palmer BW Golshan S Heaton RK Generalized cognitive impairments, ability to perform everyday tasks, and level of independence incommunity living situations of older patients with psychosis. Am J Psychiatry (2002) 159(12):2013–20. 10.1176/appi.ajp.159.12.2013 12450950

[20] Schuitevoerder S Rosen JW Twamley EW Ayers CR Sones H Lohr JB A meta-analysis of cognitive functioning in older adults with PTSD. J Anxiety Disord (2013) 27:550–8. 10.1016/j.janxdis.2013.01.001 23422492

[21] Qureshi SU Long ME Bradshaw R Pyne JM Magruder KM Kimbrell T Does PTSD Impair Cognition Beyond the Effect of Trauma? J Neuropsych Clin N (2011) 23:16–28. 10.1176/appi.neuropsych.23.1.16 21304135

[22] Aupperle RL Melrose AJ Stein MB Paulus MP Executive function and PTSD: disengaging from trauma. Neuropharmacology (2012) 62:686–94. 10.1016/j.neuropharm.2011.02.008 PMC4719148 21349277

[23] Twamley EW Allard CB Thorp SR Norman SB Hami-Cissell S Hughes-Berardi K Cognitive impairment and functioning in PTSD related to intimate partner violence. J Int Neuropsychol Soc (2009) 15:879–87. 10.1017/S135561770999049X 19703324

[24] Vasterling JJ Duke LM Brailey K Constans J II Allain AN Jr Sutker PB Attention, learning, and memory performances and intellectual resources in Vietnam veterans: PTSD and no disorder comparisons. Neuropsychology (2002) 16(1):5–14. 10.1037//0894-4105.16.1.5 11853357

[25] Zen AL Whooley MA Zhao S Cohen BE Post-traumatic stress dis-order is associated with poor health behaviors: findings from the heart and soul study. Health Psychol (2011) 31:194–201. 10.1037/a0025989 22023435 PMC3295904

[26] Kessler RC Frank RG The impact of psychiatric disorders on work loss days. Psychol Med (1997) 27:861–73. 10.1017/S0033291797004807 9234464

[27] Karney BR Crown JS Families under stress: an assessment ofdata, theory, and research on marriage and divorce in the military [Mono-graph]. National Defense Research Institute, RAND Corporation. Santa Monica, California: RAND Corporation, MG-599-OSD, 2007 (2007). Available at: https://www.rand.org/pubs/monographs/MG599.html As of January 26, 2020.

[28] Alamiri B Garrett CN Fitzmaurice M Murphy JM Gilman SE Neurological Soft Signs and Cognitive Performance in Early Childhood. Dev Psychol (2018) 54(11):2043–52. 10.1037/dev0000566 PMC6202138 30265034

[29] Chan RC Rao H Chen EE Ye B Zhang C The neural basis of motor sequencing: An fMRI study of healthy subjects. Neurosci Lett (2006) 398:189–94. 10.1016/j.neulet.2006.01.014 16469446

[30] Dazzan P Morgan KD Chitnis X Suckling J Morgan C Fearon P The structural brain correlates of neurological soft signs in healthy individuals. Cereb Cortex (2006) 16:1225–31. 10.1093/cercor/bhj063 16251504

[31] Herold CJ Duval CZ Lässer MM Schröder J Neurological soft signs (NSS) and cognitive impairment in chronic schizophrenia. Schizophr Res Cognit (2019) 16:17–24. 10.1016/j.scog.2018.12.002 30671351 PMC6305804

[32] Heinrichs DW Buchanan RW Significance and meaning of neurological signs in schizophrenia. Am J Psychiatry (1988) 145:11–8. 10.1176/ajp.145.1.11 3276226

[33] Boks MPM Russo S Knegtering R van den Bosch RJ The specificity of neurological signs in schizophrenia: a review. Schizophr Res (2000) 43:109–16. 10.1016/s0920-9964(99)00145-0 10858629

[34] Baş TO Poyraz CA Baş A Poyraz BÇ Tosun M The impact of cognitive impairment, neurological soft signs and subdepressive symptoms on functional outcome in bipolar disorder. J Affect Disord (2015) 174:336–41. 10.1016/j.jad.2014.12.026 25545601

[35] Goswami U Gulrajani C Varma A Sharma A Ferrier IN Young PAH Soft neurological signs do not increase with age in euthymic bipolar subjects. J Affect Disord (2007) 103(1-3):99–103. 10.1016/j.jad.2007.01.009.2006 17367868

[36] Colom R Jung RE Haier RJ Distributed Brain Sites for the g-Factor of Intelligence. NeuroImage (2006) 31:1359–13.65. 10.1016/j.neuroimage.2006.01.006 16513370

[37] Luders E Narr KL Thompson PM Toga AW Neuroanatomical Correlates of Intelligence. Intelligence (2009) 37(2):156–63. 10.1016/j.intell.2008.07.002 PMC2770698 20160919

[38] Chan RCK Xu T Heinrichs RW Yu Y Wang Y Neurological Soft Signs in Schizophrenia: A Meta-analysis. Schizophr Bull (2010) 36(6):1089−104. 10.1093/schbul/sbp011 19377058 PMC2963054

[39] Gurvits TV Gilbertson MW Lasko NB Tarhan AS Simeon D Macklin ML Neurologic Soft Signs in Chronic Posttraumatic Stress Disorder. Arch Gen Psychiatry (2000) 57(2):181−6. 10.1001/archpsyc.57.2.181 10665621

[40] Gurvits TV Carson MA Metzger L Croteau HB Lasko NB Orr SP Absence of selected neurological soft signs in Vietnam nurse veterans with post-traumatic stress disorder. Psychiatry Res (2002) 110(1):81−5. 10.1016/S0165-1781(02)00026-4 12007596

[41] Marionneau A Servant M-L et Albaret J-M Échelle Psychomotrice d’évaluation des Signes neurologiques Doux (EPSID) [Neurological Soft Signs Scale]. In: Giromini F Albaret J-M Scialom P, editors. Manuel d’enseignement de psychomotricité. Paris: De Boeck-Solal (2018). p. 187–200.

[42] Ashbaugh AR Houle-Johnson S Herbert C El-Hage W Brunet A Psychometric Validation of the English and French Versions of the Posttraumatic Stress Disorder Checklist for DSM-5 (PCL-5). PloS One (2016) 11(10):e0161645. 10.1371/journal.pone.0161645 27723815 PMC5056703

[43] Tennant R Hiller L Fishwick R Platt S Joseph S Weich S The Warwick-Edinburgh Mental Well-being Scale (WEMWBS): development and UK validation. Health Qual Life Outcomes (2007) 5:63. 10.1186/1477-7525-5-63 18042300 PMC2222612

[44] Trousselard M Canini F Dutheil F Claverie D Fenouillet F Naughton G Investigating well-being in healthy population and schizophrenia with the WEMWBS. Psychiatry Res (2016) 245:282–90. 10.1016/j.psychres.2016.08.050 27565700

[45] Keyes CLM Promoting and protecting mental health as flourishing: A complementary strategy for improving national mental health. Am Psychol (2007) 62(2):95–108. 10.1037/0003-066X.62.2.95 17324035

[46] Lecrubier Y Weiller E Hergueta P Bonora L Lepine J The Mini International Neuropsychiatric Interview (M.I.N.I.), a short diagnostic interview: Reliability and validity according to the CIDI. Eur Psychiatry (1992) 12:232–41.

[47] Dunn OJ Multiple Comparisons Using Rank Sums. Technometrics (1964) 6(3):241–52. 10.1080/00401706.1964.10490181

[48] Kruskal W Wallis W Use of Ranks in One-Criterion Variance Analysis. J Am Stat Assoc (1952) 47(260):583–621. 10.2307/2280779

[49] Hori H Kim Y Inflammation and post-traumatic stress disorder. Psychiatry Clin Neurosci (2019) 73:143–53. 10.1111/pcn.12820 30653780

[50] Speer K Upton D Semple S McKune A Systemic low-grade inflammation in post-traumatic stress disorder: a systematic review. J Inflammation Res (2018) 11:111–21. 10.2147/JIR.S155903 PMC5868606 29606885

[51] Miller MW Lin AP Wolf EJ Miller DR Oxidative Stress, Inflammation, and Neuroprogression in Chronic PTSD. Harv Rev Psychiatry (2018) 26(2):57–69. 10.1097/HRP.0000000000000167 29016379 PMC5843493

[52] Miller MW Sadeh N Traumatic stress, oxidative stress and posttraumatic stress disorder: neurodegeneration and the accelerated-aging hypothesis. Mol Psychiatry (2014) 19(11):1156–62. 10.1038/mp.2014.111 PMC4211971 25245500

[53] Carletto S Borsato T Neurobiological correlates of post-traumatic stress disorder: A focus on cerebellum role. Eur J Trauma Dissoc (2017) 1:153–7. 10.1016/j.ejtd.2017.03.012

[54] Holmes SE Scheinost D DellaGioia N Davis MT Matuskey D Pietrzak RH Cerebellar and Prefrontal Cortical Alterations in PTSD: Structural and Functional Evidence. Chronic Stress (2018) 2:1–11. 10.1177/2470547018786390 PMC6054445 30035247

[55] Lee HW Arora J Papademetris X Tokoglu F Negishi M Scheinost D Altered functional connectivity in seizure onset zones revealed by fMRI intrinsic connectivity. Neurology (2014) 83(24):2269–77. 10.1212/WNL.0000000000001068 PMC4277677 25391304

[56] Botez Cerebellum MI Ramachandran VS eds. Encyclopedia of Human Behavior Vol. p New York: Academic Press (1999). p. 2002.

[57] Schmahmann JD Caplan D Cognition, emotion and the cerebellum. Brain (2006) 129:290–2. 10.1093/brain/awh729 16434422

[58] Nicholson AA Rabellino D Densmore M Frewen PA Paret C Kluetsch R The neurobiology of emotion regulation in posttraumatic stress disorder: Amygdala downregulation via real-time fMRI neurofeedback. Hum Brain Mapp (2017) 38(1):541–60. 10.1002/hbm.23402 PMC6866912 27647695

[59] Patel R Spreng RN Shin LM Girard TA Neurocircuitry models of posttraumatic stress disorder and beyond : a meta-analysis of functional neuroimaging studies. Neurosci Biobehav Rev (2012) 36(9):2130–42. 10.1016/j.neubiorev.2012.06.003 22766141

[60] Schutter DJ Van Honk J The cerebellum on the rise in human emotion. Cerebellum (2005) 4:290–4. 10.1080/14734220500348584 16321885

[61] Lange I Kasanova Z Goossens L Leibold N De Zeeuw C II van Amelsvoort T The anatomy of fear learning in the cerebellum: a systematic meta-analysis. Neurosci Biobehav Rev (2015) 59:83–91. 10.1016/j.neubiorev.2015.09.019 26441374

[62] Ernst TM Brol AE Gratz M Ritter C Bingel U Schlamann M The cerebellum is involved in processing of predictions and prediction errors in a fear conditioning paradigm. ELife (2019) 8:e46831. 10.7554/eLife.46831 31464686 PMC6715348

[63] Utz A Thürling M Ernst TM Hermann A Stark R Wolf OT Cerebellar vermis contributes to the extinction of conditioned fear. Neurosci Lett (2015) 604:173–7. 10.1016/j.neulet.2015.07.026 26219987

[64] Fan L Tang Y Sun B Gong G Chen ZJ Lin X Sexual dimorphism and asymmetry in human cerebellum: An MRI-based morphometric study. Brain Res (2010) 1353:60–73. 10.1016/j.brainres.2010.07.031 20647004

[65] Osuch EA Benson B Geraci M Podell D Herscovitch P McCann UD Regional cerebral blood flow correlated with flashback intensity in patients with posttraumatic stress disorder. Biol Psychiatry (2001) 50(4):246–53. 10.1016/s0006-3223(01)01107-6 11522258

[66] Pissiota A Frans O Fernandez M von Knorring L Fischer H Fredrikson M Neurofunctional correlates of posttraumatic stress disorder: a PET symptom provocation study. Eur Arch Psychiatry Clin Neurosci (2002) 252(2):68–75. 10.1007/s004060200014 12111339

[67] Baldaçara L Jackowski AP Schoedl A Pupo M Andreoli SB Mello MF Relationship between structural abnormalities in the cerebellum and dementia, posttraumatic stress disorder and bipolar disorder. Dement Neuropsychol (2012) 6(4):203–11. 10.1590/S1980-57642012DN06040003 PMC5619331 29213799

[68] Haskell SG Gordon KS Mattocks K Duggal M Erdos J Justice A Gender differences in rates of depression, PTSD, pain, obesity, and military sexual trauma among Connecticut War Veterans of Iraq and Afghanistan. J Womens Health (Larchmt) (2010) 19(2):267–71. 10.1089/jwh.2008.1262 PMC3052274 20109115

[69] Olff M Sex and gender differences in post-traumatic stress disorder: an update. Eur J Psychotraumatol (2017) 8(4):1351204. 10.1080/20008198.2017.1351204

[70] Hu J Feng B Zhu Y Wang W Xie J Zheng X Gender Differences in PTSD : Susceptibility and Resilience. London, UK: IntechOpen (2017). 10.5772/65287

[71] Christiansen DM Hansen M Accounting for sex differences in PTSD: A multi-variable mediation model. Eur J Psychotraumatol (2015) 6(1):26068. 10.3402/ejpt.v6.26068 25604705 PMC4300366

[72] Goos LM Silverman I Sex related factors in the perception of threatening facial expressions. J Nonverbal Behav (2002) 26(1):27–41. 10.1023/A:1014418503754

[73] McClure EB A meta-analytic review of sex differences in facial expression processing and their development in infants, children, and adolescents. Psychol Bull (2000) 126(3):424–53. 10.1037/0033-2909.126.3.424 10825784

[74] Frijling JL Preventing PTSD with oxytocin: Effects of oxytocin administration on fear neurocircuitry and PTSD symptom development in recently trauma-exposed individuals. Eur J Psychotraumatol (2017) 8(1):1302652. 10.1080/20008198.2017.1302652 28451068 PMC5400019

[75] Inslicht SS Metzler TJ Garcia NM Pineles SL Milad MR Orr SP Sex differences in fear conditioning in posttraumatic stress disorder. J Psychiat Res (2013) 47(1):64–71. 10.1016/j.jpsychires.2012.08.027 23107307 PMC3806498

[76] Diedrichsen J Balsters JH Flavell J Cussans E Ramnani N A probabilistic MR atlas of the human cerebellum. NeuroImage (2009) 46(1):39–46. 10.1016/j.neuroimage.2009.01.045 19457380

[77] Xin J Zhang Y Tang Y Yang Y Brain Differences Between Men and Women: Evidence From Deep Learning. Front Neurosci (2019) 13:185. 10.3389/fnins.2019.00185 30906246 PMC6418873

[78] Kaczkurkin AN Raznahan A Satterthwaite TD Sex differences in the developing brain: insights from multimodal neuroimaging. Neuropsychopharmacol (2019) 44:71–85. 10.1038/s41386-018-0111-z PMC6235840 29930385

